# 

**Published:** 1935

**Authors:** 


					Vol. LH. No. 197.
m , ' H A
Jifcfv
_ ?? miHI
AUTUMN, 1935.
.
The Bristol
# ^ " _
-JJ ^V ?' : : ? -"-? ?" SSJr i
lv -?r. ? ? . - Uj
. \ ? .
PtTBXXSHKD TTNDEK THE AUSPICES OF
THE BRISTOL MEDICO-CHIRURGICAL SOCIETY.
Editor: J. A. NIXON, C.M.G., M.D., F.R.C.P.
mh, ? .. '??* ? ? ? if ?i. <Jwy .
* ' "> '*'? '?'???* . '-*h
WITH WHOM ABB ASSOCIATED
? r*.y . ;- ':pi;Kr: ' :.p, ? ' Cv- '?
E. W. HEY GROVES, D.So., M.S., F.R.C.S.
A. E. ILES, O.B.E., M.B., B.S., F.R.C.S,
A. RENDLE SHORT, M.D., B.S., B.Sc., F.R.C.S.
E. WATSON-WILLIAMS, M.O., Ch.M., F.R.C.S., Aasistanl Editor.
Editorial Secretary: A. L. FLEMMING, M.B., Cb.B.
j
?
*2 S-: :? .. - ,
' ' . - ? ?? .
i  _   Attitr
BRISTOL: J. W. ARROWSMITH LTD., 11 QUAY STREET
? t 1 IS T    . T XXT AoTinnrciirimr /T.nwtinw^ T/rr>
London: J. W. Abrowsmith (London) Ltd.
Fui/wood House, Fuxwood Place, High Holbobn, W.C. 1
I Price Three Shillings net.
Ik
l*WJIT*i> /ft Great Britain

				

